# Generations and political change

**DOI:** 10.1080/01402382.2025.2449794

**Published:** 2025-01-13

**Authors:** Wouter van der Brug, Sylvia Kritzinger

**Affiliations:** a Department of Political Science, University of Amsterdam, The Netherlands; b Department of Government, University of Vienna, Austria

**Keywords:** Generations, political socialisation, APC-models, political change, de- and realignment

## Abstract

The Symposium on Generations and Political Change brings together articles that focus on the role of generational differences in political behaviours and attitudes. As younger generations will be gradually replacing the older generations, generational difference informs us about potential political changes that lie ahead. This introductory article discusses the contributions that the articles make to the wider (and rapidly expanding) field of political socialisation and deduce from there some avenues for future research and data requirements.

The political attitudes and behaviours of young people differ substantially from those of older generations. For instance, young people are more supportive of European unification than older citizens and more likely to have voted ‘remain’ in the Brexit referendum in the UK (e.g., Down and Wilson 2013; Hobolt 2016). They are more willing to support measures to protect the environment and to reduce global warming, and they are more likely to vote for Green parties (e.g., Goerres 2008; Lichtin *et al.* 2023). Moreover, young people are at the forefront of protest movements, such as Extinction Rebellion and Black Lives Matter (e.g., Boulianne *et al.* 2020), but they are less likely to turn out at elections (e.g., Franklin 2004; Grasso 2014; Marien *et al.* 2010). Based on these observations, one might expect that there will be large political changes ahead as these new generations will gradually replace the generations before them. If their behavioural patterns and attitudes remain stable, we can expect turnout to decrease, but protest to increase and public opinion to shift towards more social liberal positions. However, as generational replacement is a slow and gradual process, the associated changes can only occur gradually as well. Changes have been compared to a ‘diesel engine’: it gets off to a very slow start, but once going, it keeps running for a very long time (Van der Brug and Franklin 2018).

However, while there are good theoretical reasons to expect generations to differ from each other, some of the observable differences between age groups might be simply the result of life-cycle effects. After all, has it not always been the case that young people are more progressive and more likely to protest than older people? If the age differences are ‘just’ life-cycle effects, the active and progressive youngsters can be expected to become more passive and conservative as they grow older. In that case, generational replacement will not lead to any meaningful political changes. In fact, the demographic changes resulting from the ageing of the populations of many advanced democracies would then lead to more conservatism at the aggregate level. In order to acquire a good understanding of the nature of political change, one should thus not only take into account differences in term of differing cohorts, but one should also try to disentangle potential generational differences from life-cycle effects.

These insights are neither very novel nor original. Inglehart’s (1977) famous theory of the ‘Silent Revolution’ is based on the idea that the post-war generation that was socialised in a period of peace and prosperity would be more likely to prioritise postmaterialist values than the generations before them who experienced WWII and experienced more hardship when they were young. Inglehart’s theory was based on the idea that political attitudes and behavioural habits that are acquired early in life during the most ‘formative years’ (roughly between 15 and 25, also called the ‘impressionable years’) tend to remain rather stable afterwards. This is due to the notion that people tend to get ‘set in their ways’ as they grow older (Franklin 2004).

Nevertheless, the theoretical underpinnings of political socialisation and of generational differences have attracted much renewed research interest over the past decade.^1^

Studies have been published about political socialisation in the area of turnout and other forms of mobilisation (e.g., Dinas 2012; Franklin 2004; Grasso 2014; Marien *et al.* 2010), political attitudes (e.g., Down and Wilson 2013; Neundorf 2010), party preferences and party choice (e.g., Dassonneville 2013; Dinas 2014; Goerres 2008; Grasso *et al.* 2019; Maggini 2016; Tilley and Evans 2014) and the drivers of party choice (e.g., Gougou and Mayer 2013; Van der Brug 2010; Van der Brug and Rekker 2021; Wagner and Kritzinger 2012). So, why this renewed interest, if the underlying theories have been around for so long?

One reason is probably that scholars want to know to what extent generational replacement contributes to the political changes that we observe since political attitudes and patterns of party choice may differ between generations. Another reason is that more and longer time series data are now available, which are needed to disentangle the fully multicollinear effects of age (life-cycle), period and cohort (generation) (APC). To estimate an APC-model a time frame of minimally two decades would be needed to arrive at robust estimates. Some of the larger trend studies, like the Comparative Studies of Electoral Systems (CSES), the European Social Survey (ESS) and the European Elections Studies (EES), have now been running for more than 25 years, and furthermore Household Panel Data have become available, allowing researchers to employ these data sources for new research questions in the field of political socialisation – as it is also the case with several contributions to this Symposium. Next to data availability also methodological progress and fast computers have facilitated research in this field.

Each of the articles in this Symposium makes important contributions to the literature on political socialisation. Three of the four articles present APC models to study questions pertaining to electoral processes. A fourth article employs panel data to study changes in attitudes among adolescents to study the socialising effect of education. Below we first discuss briefly the main findings of these different articles. We then reflect upon their contributions to the field, and we end by identifying some remaining gaps in the literature.

## The main findings of the symposium

How does generational replacement contribute to patterns of dealignment and realignment? This general question is taken up by two articles in the symposium both employing APC-analysis for this purpose (Jocker *et al.* 2024; Mitteregger 2024). Mitteregger (2024) explores dealignment and realignment by focusing on voters’ ‘consideration sets’ in a two-stage model of party choice. According to this model, voters in multiparty systems have a limited set (of usually) ideologically similar parties that they might consider voting for. From the large subset of parties, voters will select a few parties into their ‘consideration set’ and base their ultimate voting decision by comparing (in greater detail) the parties in this consideration set. By means of APC-modelling, Mitteregger first shows that the newest generations tend to have a larger number of parties in their consideration set, which he interprets as a sign of dealignment as voters who are very strongly aligned to a specific party will usually not consider many other parties, resulting into a small consideration set. He concludes that younger generations thus display less partisan alignment than the older generations. Meanwhile, to explore a potential realignment, he focuses on the ideological differences between the parties the two generations contemplate in the consideration set. Interestingly, the parties in the consideration sets of the younger generations differ more on the socio-economic dimension than on the socio-cultural dimension. This is not the case for older generations. This suggests realignment along the socio-cultural dimension, as newer generations tend to restrict their consideration set mainly to parties that are ideologically close on socio-cultural issues. Meanwhile, a process of dealignment is taking place on the socio-economic dimension.

The article by Jocker *et al.* (2024) focuses upon a different type of link between parties and voters, which they call ‘issue alignment’. Issue alignment is defined as the strength of the relationship between party choice and issue preferences. On the basis of political socialisation theory, they expect that younger generations align more strongly on ‘emerging issues’ such as immigration, European integration, and the environment, compared to older generations who were socialised before these issues became salient and gained political significance. Using ESS and Chapel Hill Expert Survey data, and again by means of APC-modelling, their results reveal that the newer generations are indeed more issue-aligned on immigration, LGBTQ+-rights, the environment and left–right placement than the generations before them – thus being in line with Mitteregger’s findings –, but this is not case on European integration and income redistribution. They also find over-time increases in issue alignment for three of the six issues that they study. Somewhat unexpectedly, their study does not indicate that older generations are more strongly aligned on the issue of income redistribution, but on the other hand there is no issue on which alignment declines over time. The overall conclusion they draw is that Western Europe experiences a partial trend towards *more* issue alignment, particularly on the issue of immigration, and that generational replacement is an important driver of this trend.

In line with Jocker *et al.* and Mitteregger, Li (2024) also finds clear generational differences when focusing on the difference in turnout between national and European Parliament (EP) elections. Traditionally, turnout in EP elections is substantially lower than in national elections. Yet, in the 2019 EP elections, turnout was higher than in previous ones. This was especially the case for the youngest voters, which is a group that traditionally is least likely to participate in elections. Li’s APC-models across EU member states over several decades using CSES and EES data reveal diverging generational participatory patterns between EP and national elections. While recent cohorts are increasingly less likely to participate in national elections than earlier ones, the youngest cohort becomes somewhat more likely to participate in EP elections than those born in the 1970s. Despite country-level differences, pooled APC-analyses identify a reversing generational participatory pattern in EP elections and a narrowing turnout gap between EP and national elections across younger generations. These results provide critical insight both into the ‘negative’ effect of generational replacement on aggregate turnout and the type of election, and have furthermore important implications for the future trends of democratic deficit and participatory inequalities in national and European politics.

Finally, using British and Swiss Household Panel Data, Ares and Van Ditmars (2024) examine how socialisation of adolescents contributes to class-based differences in political attitudes. Employing panel data, they study attitudes already at a relatively young age and trace these at different stages in people’s lives. This enables them to assess at what stage of individuals’ lives class differences begin to emerge: in early adulthood, in the transition to one’s first job, or in the class of destination? Their analyses show that differences in political attitudes between (future) social class groups exist already in early adulthood. Thus, the process of political development and learning takes place already relatively early in life. However, and most importantly, attitudinal differences continue to consolidate as people enter the workplace and reach their main class of destination, indicating further socialisation into the attitudes of those people an individual interacts with professionally. This research advances current and historical debates about social class as a relevant milieu of political socialisation and public opinion formation, and connects it to the educational cleavage based on social class and the importance of early political socialisation.

## Lessons learned and avenues for future research

Taking a helicopter view of the articles in this Symposium, the evidence presented may seem somewhat contradictory with regards to political change. Perhaps the most obvious contradiction is that all three studies based on APC-analysis find evidence for generational differences (Jocker *et al.* 2024; Li 2024; Mitteregger 2024), while the study by Ares and Van Ditmars (2024) suggests that attitudinal differences exist already largely at a young age. However, if differences exist already before the impressionable years, one might wonder whether the early adulthood socialisation is really as important as it is assumed by some scholars. More precisely, differences in political attitudes that exist already quite early in life are most likely the result of parental socialisation and the ‘milieu’ in which people grew up, leading to no or small differences between generations. So, how do we explain these seemingly different findings? In the following we present some readings of the findings combined with ideas for future research agendas.

First, patterns observed by social scientists are never deterministic, nor do we expect that the phenomenon we want to explain has just one single cause. On the one hand, there are factors that reduce differences between generations, like the influence of parents and the social milieu in which children grow up. On the other hand, there are factors that produce differences between generations, such as events happening during one’s impressionable years, which may have a lasting impact on one’s outlook on the world. What we observe is probably a mix between socialising factors that reduce generational differences (and thus produce stability), and those that increase such differences (and thus produce change).

A second aspect to consider is that the studies presented in this Symposium focus on different dependent variables. When it comes to very basic values about fairness or prejudice against certain groups of citizens, like ethnic minorities or people with different religious beliefs, children might have already formed these attitudes at a very young age (see for instance, van Deth *et al.* 2011) with parental socialisation more likely playing a crucial role. Yet, in most countries people only obtain the right to vote when they turn 18, and few people take an interest in (party) politics at a very young age (e.g., Bergh 2013). Thus, also the development of partisan attitudes may take place around that age (Bartels and Jackman 2014; Rekker *et al.* 2019; Schuman and Rodgers 2004), with young citizens around that time relying less upon their parents and many even rebelling against (the generation of) their parents. When focusing on party preferences or electoral behaviour, we may thus expect to find smaller influences of the parents than when we investigate other types of attitudes and behaviours. Again, this does not mean that parents do not influence the party preferences of their children (e.g., Achen 2002; Coffé & Voorpostel 2010; Durmuşoğlu *et al.* 2023; Hooghe & Boonen 2015), but generational differences are plausibly larger than they are in other areas.

The findings regarding dealignment and realignment also reveal some contradictions. Mitteregger finds clear evidence of *partisan dealignment* across generations, whereas Jocker *et al.* find that younger generations become *more aligned on issues*. While these findings seem inconsistent, this is not necessarily the case. Someone who is strongly attached to one single party and who will subsequently always vote for such a party, will be viewed as scoring high on partisan alignment. This speaks to the origin of how we think about (partisan) alignment. As older generations are more likely to be aligned with one single party than younger birth cohorts (Mitteregger 2024), if ‘their’ party gives strong signals about its position on a certain (newly politicised) issue, they may be persuaded to also adopt that same position given their strong partisan attachment. In such a scenario, the members of older generations would also be strongly aligned on issues. However, strong partisan alignment might also hinder issue alignment, if feelings of solidarity and allegiance to a specific party leads one to keep supporting the party, even if one disagrees with the party on specific issues – or is not aware of its position on newly politicised issues. If that happens, citizens with strong partisan alignments, may support a party even if it is not the closest to their position on several issues, and hence score lower on issue alignment. Meanwhile, younger generations are more strongly aligned on issues than generations before them but score lower on partisan alignment (Jocker *et al.* 2024). If we combine these two perspectives, the most likely explanation is that partisan dealignment provides room for more issue alignment, while strong partisan alignment may undermine issue alignment.

Turning now to the common patterns observed in the articles presented in the Symposium, we can observe common generational differences at the economic and cultural ideological dimension across generations. Based on the time frames analysed, younger generations seem to have a stronger focus on cultural aspects both reflected in issue alignment and the analysed considerations sets. Meanwhile, for older generations the economic dimension seems to be an important structuring element. This seems also to be reflected in the different turnout behaviour of younger cohorts. The turnout gap between European and national elections is smaller among more recent birth cohorts than among older ones. Issues of European integration seem to be of greater concern to the most recent generations. However, it remains to be seen how the underlying early social class socialisation affects future generational development both on cultural and economic issues. What is more, it remains to be seen how the various economic crisis situations – so-called period effects – such as the economic and financial crisis in 2009 or the recent high inflation may cause shifts in issue alignments both within and across generations. For instance, one could speculate that economic issues are becoming more important among younger generations, particularly in southern European countries that experienced high youth unemployment after the financial crisis, changing our current notion of issue and partisan alignment.

Overall, if we review the Symposium as a whole, the studies confirm that generational replacement is an important driver of political change. This basically means that we are very likely to see the dawning of certain political changes that will become increasingly visible in the future. The main ones are that first partisan dealignment will increase, while voters become more aligned on (cultural) issues even though we may experience shifts in the substance of these issue alignments towards economic aspects. Second, the gap in turnout between EP elections and national elections will decrease in seise, while third the political socialisation remains closely tied to social class.

Having said this, in general there is one big issue hanging over APC-studies: to separate the effects of age, period and cohort, we need to impose some very strong restrictions on the models. One of these restrictions is that we have to assume that life-cycle effects are the same for each generation and each time period. Yet, there are good reasons to doubt the validity of this assumption. If society changes, people will not just have formative experiences in the period that political scientists consider the most impressionable years (roughly 15 till 25), but maybe also in the years before and after this period. In some periods, life cycle effects could unfold differently. For instance, in the 1950s, first time voters in the US entered the electorate with quite high levels of party identification. Within this generation, the proportion of voters identifying with a party increased only slightly over the next decades. Each generation thereafter has entered the electorate with fewer party-identifyers, but over the course of decades, the percentage nevertheless also has increased over time. In other words, while the oldest generation was socialised by their parents to adopt their party-ID, next generations formed a party identification at a later age (e.g., Van der Brug and Franklin 2018). In this case, the life-cycles patterns seem thus to be different over time. Serra (2023) provides another example arguing that newer generations leave their parents’ home at a later age, also changing certain behavioural patterns. For instance, in Britain home ownership is related to voting for the Conservative party, but if people buy a home at a later phase in their lives, life-cycle patterns are bound to be different as well. These two examples outline interesting avenues for future research that are not yet very common in our field.

The study by Ares and Van Ditmars opens up for another important area for future research: a focus on the political socialisation of young children. There is increasing evidence that political attitudes are passed on between parents and children already at a quite young age. When entering high school we can already see differences in some political attitudes between groups of children, which resemble those of their parents. While these opinions may become more pronounced as they grow up, there are important developments taking place at a younger age that we normally do not observe. Measuring political attitudes of children is certainly challenging, but as political socialisation seems to already occur at a young age, future research focusing more intensively on the micro-level mechanisms of childhood socialisation seems more than worthwhile.

However, one great challenge hangs over all these future research avenues. Our field will need particular data to engage in detail in this type of research to understand political changes better over time as well as across countries. We need both longer running longitudinal studies and panel studies to be able to assess how life-cycle patterns (do not) change between generations. Having such data at hand and collecting it on a continuous basis, we can both circumvent the outlined problems intrinsic in current APC-models and make use of rich data collection to engage in greater detail with research questions of political change – and thus potentially of changing societies.

## Note

1 A personal ‘qualitative’ observation supports this. Over a decade ago, we edited a special issue on the topic, which was the result of an ECPR joint session workshop (Van der Brug and Kritzinger 2012). When we organized a similar workshop in 2023, the one that was at the basis of this Symposium, we had more than twice the number of good proposals as we had back in 2010.
